# An Infant with Asymptomatic Vitamin D Intoxication: A Prolonged and Sustainable Recovery

**DOI:** 10.1155/2022/7072815

**Published:** 2022-02-27

**Authors:** Ibrahim Al Alwan, Nouf Al Issa, Yousef Al Anazi, Khalid Al Noaim, M. Zulf Mughal, Amir Babiker

**Affiliations:** ^1^College of Medicine, King Saud Bin Abdulaziz University for Health Sciences, Riyadh, Saudi Arabia; ^2^Endocrine Division, Department of Pediatrics, King Abdullah Specialized Children Hospital, Riyadh, Saudi Arabia; ^3^King Abdullah International Medical Research Centre, Ministry of the National Guard Health Affairs, Riyadh, Saudi Arabia; ^4^Department of Pediatrics, College of Medicine, Majmaah University, Majmaah, Saudi Arabia; ^5^Department of Pediatrics, College of Medicine, King Faisal University, Al-Ahsa, Saudi Arabia; ^6^Department of Paediatric Endocrinology, Royal Manchester Children's Hospital, Manchester University NHS Foundation Trust, Manchester, UK; ^7^Faculty of Biology, Medicine and Health, University of Manchester, Manchester, UK

## Abstract

Vitamin D intoxication (VDI) usually develops due to inappropriate use of vitamin D in high doses by the families of infants with complaints suggestive of vitamin D deficiency such as delayed teething, knock knees, or delayed walking. We present here an experience of treating an infant with asymptomatic VDI that had a prolonged course of recovery and a sustainable level of vitamin D over a follow-up period of 2.5 years. In our patient, vitamin D started to drop steadily after a month of stopping vitamin D supplements but not to a normal level. It reached an acceptable level only after six months. This case emphasizes the importance of educating parents about the empirical use of vitamin D over the counter, assessing the baseline level of serum vitamin D level prior to initiation of treatment and highlights the value of verifying additional dietary sources of vitamin D or oral supplements in patient's history.

## 1. Background

Vitamin D exists in three inactive forms, that is, cholecalciferol, ergocalciferol, and 7-dehydrocholesterol. Cholecalciferol is found in animal-sourced foods, whereas ergocalciferol mainly comes from plant sources and fortified foods. During sun exposure, 7-dehydrocholesterol is produced endogenously in the skin; then, the above three inactive forms are converted to 25-hydroxyvitamin D (25(OH)D) in the liver. A final hydroxylation to 1,25-dihydroxyvitamin D (1,25-(OH)2D) occurs in the kidney to the active form of vitamin D [1]. The active form binds to the vitamin D receptor (VDR) to increase intestinal calcium and phosphate absorption [2, 3].

Serum 25-hydroxyvitamin D reflects an individual's vitamin status. Vitamin D level >50 nmol/L is considered as vitamin D sufficiency, while, levels between 30 to 50 nmol/l are considered as vitamin D insufficiency and <30 nmol/l is vitamin D deficiency. Physiologically, the body maintains serum 25(OH)D concentration within a narrow range of 75–220 nmol/L across vitamin D supplies through a homeostatic process [4]. That includes critical points of metabolism that controls the level of 25(OH)D through catabolism of 25(OH)D by the liver with execration of the excess into the bile and in tissues throughout the body by the side-chain cleavage pathway into the inactive form of 24,25(OH)D [5].

A routine use of vitamin D supplements guided by physician prescription is rationalized in children with a high risk of vitamin D deficiency, such as breastfed babies, lack of exposure to sunlight, having a darker complexion, obesity, difficulty in absorbing fat in the diet, or gastric bypass surgery as well as in osteoporosis or other metabolic bone diseases [6]. However, the easy accessibility of vitamin D to patients over the counter and the empirical prescription by doctors, given its importance in general wellbeing as above, are risk factors for overdose and intoxication [7, 8]. More recently, there has been increased interest in the assessment of vitamin D level during pregnancy and the effect of vitamin D deficiency on fetus and baby [9]. However, VDI is as important as vitamin D deficiency as there is no consensus on when to start vitamin D supplementation, the optimum dose, and duration of treatment as well as the optimum level vitamin D during pregnancy [10].

VDI usually develops due to inappropriate use of vitamin D in high doses by the families of infants with complaints suggestive of vitamin D deficiency such as a delay in teething or knock knees in lower limbs and delayed walking. Other causes of VDI include empirical prescription of vitamin D supplements by health care providers before a precise diagnosis of vitamin D deficiency [11]. Estimated tolerable upper limits of vitamin D by the American Academy of Pediatrics per age are 1000 IU/day for 0-1-year-old, 2500 IU/day for 1–3 years old, 3000 IU/day for 4–8 years old, and 4000 IU/day for nine years old and above [12]. Patients with VDI usually present with symptoms of hypercalcemia such as polyuria and polydipsia with a decrease in appetite, weight loss, and gastrointestinal manifestation as nausea, vomiting, and constipation; they might also present with dehydration and seizure in severe cases [9, 10]. In VDI, high concentrations of either free 1,25(OH)2D, or 25-hydroxyvitamin D leads to hypercalcemia by intestinal calcium absorption and bone resorption [13]. Hypercalcemia increases the load in the kidney due to calcium excretion in the distal tubules, causing hypercalciuria. Persistently elevated serum calcium concentration impairs the concentrating ability of the kidneys and causes polyuria and dehydration. It can also result in nephrocalcinosis [9].

Vitamin D intoxication (VDI) is rare but probably not unusual, with a recent increase in its reporting [11]. Overdose and intoxication can occur because of prescribing, manufacturing, or formulation errors [14]. Patients and physicians should be more aware of the potential dangers of vitamin D overdose. According to the American Academy of Pediatrics, serum 25-hydroxyvitamin D (25(OH)D) level above 250 nmol/L (100 ng/ml) is considered as hypervitaminosis D. In contrast, serum 25(OH)D level above 375 nmol/L (150 ng/ml) is associated with vitamin D intoxication (VDI) [15].

We present here an experience of treating an infant with asymptomatic VDI that had a prolonged course of resolution and sustainable level of vitamin D over a lengthy period of follow-up of 2.5 years, compared with previously reported cases.

## 2. Case Presentation

An eleven-months-old girl, a full-term baby that was an outcome of cesarean section with a birth weight of 2.67 kg, had grade 1 hydronephrosis in antenatal care. She was discharged with her mother in good condition. Mother was healthy with a serum 25(OH)D level of 30.3 nmol/l in the second trimester, indicating insufficient vitamin D. She was only on ferrous sulfate but not on vitamin D supplements.

During the child's routine visit at the age of 11 months to the well-baby clinic for vaccination, she grew well and was asymptomatic with a height of 72.5 cm (25–50th centile) and a 9.2 kg (50th centile) weight. Her vital signs were acceptable. She was alert and well-hydrated, vitally stable afebrile on examination with acceptable growth parameters for her age. The other examination was unremarkable, and there was no alopecia.

**Figure 1:**
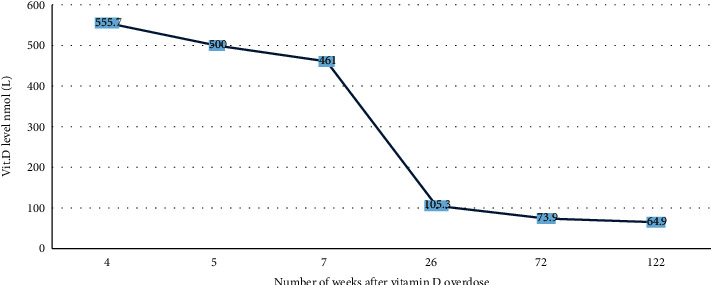
Vitamin D levels over 2.5 years of follow-up.

The primary physician requested routine laboratory tests due to the mother's concerns of delayed teething and hair loss. It showed a serum 25(OH)D level of 555 nmol/L (reference range >250 nmol/L toxicity) with normal adjusted calcium and phosphate level 2.49 nmol/l, 1.77 mmol/l, respectively; she was on a supplementary formula with a multivitamin that contains 400 IU of vitamin D once daily for one month prescribed over the counter. Moreover, the mother applied an ampule of vitamin D (Steril Devit-3 I.M/oral ampule) that contains 300,000 IU on the baby's gum one month before that visit as recommended by one of her relatives. The child remained asymptomatic during follow-up, and her serum 25(OH)D level normalized to 105.3 nmol/L after 6 months of discontinuing vitamin D supplements (Figure 1). In addition, the patient had normal other chronological investigations of the bone profile including serum calcium levels (Table 1). The level of 25(OH)D remained normal after 2.5 years of discontinuing treatment despite no additional sources of vitamin D supplements or extra exposure to sunlight (Figure 1). Repeated renal ultrasound showed persistent bilateral grade 1 hydronephrosis with no nephrocalcinosis.

## 3. Discussion

Our patient exceeded the tolerable upper limits of vitamin D level for her age. However, she remained asymptomatic and maintained a normal level of 25(OH)D for 2.5 years post ingestion of a supraphysiologic dose of vitamin D.

Systematic studies on vitamin D intoxication (VDI) have been conducted in a variety of different animal species. These studies showed that the plasma 25(OH)D concentrations associated with toxicity were always over 375 nmol/L [16]. For ethical reasons, no systematic experimental studies have examined VDI in humans. However, numerous anecdotal reports over the years have described accidental VDI. Jones, in a review of pharmacokinetics of vitamin D toxicity, found that all reported cases with overt symptoms of VDI had a concentration of 25(OH)D well above the normal range at 710–1587 nmol/L, with several patients exhibiting values consistently around 750 nmol/L [16]. Supporting a previous conclusion by Vieth et al., Jones established that hypercalcemia only results when 25(OH)D3 concentrations have always been above 375–500 nmol/L [7, 16]. In a recent review, the Pediatric Endocrine Society assessed the risk of VDI in infants and children, and reports on VDI in infants and young children were presented with severe hypercalcemia and serum 25(OH)D level in a range of 250–670 ng/mL (625–1675 nmol/L). There is significant variability in the amount of vitamin D administered and the resulting serum 25(OH)D concentrations among these cases. Rather than a specific level of 25(OH)D, the diagnosis of VDI was based on elevated serum 25(OH)D concentrations associated with hypercalcemia or hypercalciuria, whereas serum 1,25(OH)2D levels were normal and PTH was suppressed.

Despite the randomized clinical trials with evidence that symptomatic toxicity has only been reported with serum levels of 25(OH)D at levels >500 nmol/L. To allow a large safety margin, the global consensus on nutritional rickets defined Vitamin D toxicity as hypercalcemia and serum 25(OH)D >250 nmol/L with hypercalciuria and suppressed PTH [6].

Our patient was asymptomatic with an accidental finding of a high vitamin D level exceeding 500 nmol/L a month after ingesting a single dose of oral vitamin D3 in a routine visit, with normal adjusted calcium and phosphorus levels as well as normal PTH levels. She had no prior vitamin D level reported results.

Reviewing previous literature of infants with VDI (Supplement A), we postulate that variations in the duration and frequency of the overdose could lead to variability in associated symptoms due to variable hormonal responses that control vitamin D metabolism. In previous reports, infants with symptomatic vitamin D intoxication had received daily large doses (50,000–200,000 IU of vitamin D3) over a longer duration (1–4 months) [13, 17–24]. On the other hand, those with an asymptomatic course of illness had received relatively much smaller doses (10,000–12,000 IU of vitamin D3) over a shorter duration (less than a month) before presentation [23, 24]. Moreover, it might also be important to evaluate patients with VDI, especially when asymptomatic, for polymorphism of vitamin D binding protein (DBP) and gene mutations of CYP24A1, which code for 24-hydroxylase enzyme that change vitamin D into an inactive form. Unfortunately, these tests were not performed on our patient.

Our patient, a ten-month-old child, ingested a single mega dose of vitamin D that was accidentally recognized after a month of that event. The duration of receiving toxic amounts of vitamin D supplements (a stagnant dose) and the resultant serum calcium level seem to affect symptomatology. Probably, a single nonrepeated large dose in our patient did not “soak up” DBP compared to the stagnant dose over a more extended period described in previous reports that made our patient asymptomatic. In addition, as in previous reports, the cut point of vitamin D level beyond which one would expect symptoms was 750 nmol/l, and our patient vitamin D level was less than that level [16]. Despite that a vitamin D level of 375 nmol/l was described as the critical level for VDI in previous reports, the cut points of Ca levels associated with symptoms using Spearman's correlation in that Turkish reports was found to be above 3.5 mmol/l [11, 25]. Since we do not have previous laboratory results of calcium level in our patient, she probably had have mild (2.5–2.9 mmol/l) to moderate (3.0–3.5 mmol/l) serum calcium levels before her initial presentation a month after the ingestion of vitamin D overdose. Absence or mild soaked-up effect of DBP, a vitamin D level less than 750 nmol/l, and a possible mild to moderate hypercalcemia prior to presentation could all contribute to our patient' asymptomatic course of illness.

The half-life of vitamin D3 is approximately 15 days; however, VDI might take several months to resolve even after stopping the medication because it is a lipophilic vitamin and is stored in fat [9]. In our patient, vitamin D started to drop steadily after a month of stopping vitamin D supplements but not to a normal level until after six months from the initial presentation. Last but not least, all previous reports have focused on the effect of VDI to the point of time when the patients recovered, i.e., when vitamin D level is normalized. In our patient, a follow-up of 2.5 years after recovery, i.e., a normal vitamin D level after six months of follow-up, revealed a steady level of vitamin D (around 65 nmol/l) without additional sources of vitamin D, including extra exposure to sunlight.

## 4. Conclusion

Physicians have to assess baseline blood levels of vitamin D in children before starting vitamin D supplements to avoid using higher doses that can lead to asymptomatic, yet, potentially harmful course of VDI with a prolonged recovery and sustainable high levels even without extra supplements of vitamin D in the diet as in our patient.

## Figures and Tables

**Table 1:** Biochemical values in our patients over 2 years follow-up.

Investigation	Reference value	21/03/2019 at 11 months of age	25/03/2019 at 11 months of age	3/4/2019 at 12 months of age	18/09/2019 at 17 months of age	16/08/2020 at 28 months of age	4/08/2021 at 38 months of age
Total 25-OH vit D	nmol/L	555.7	500	461	105.3	73.9	64.9
Adj Ca	2.20–2.70 mmol/L	2.49	2.47	2.33	2.46	2.42	2.28
Albumin	38–54 g/L	44	41	44	44	45	46
Ca	2.20–2.70 mmol/L	2.57	2.49	2.41	2.54	2.52	2.54
Phosphorus	1.10–1.95 mmol/L	1.74	1.81	1.62	1.88	1.82	1.61
Alkaline phosphatase	156–369 U/L	284	214	221	270	262	246

## Data Availability

The data used to support the findings of this study are included in the article and within the available supplementary information file.

## Supplementary Materials

Supplementary Materials
Figure 1: vitamin D levels per number of weeks over 2.5 years of follow-up. Table 1: biochemical values related to vitamin D metabolism in our patients over 2 years follow-up. Supplement A: clinical, biochemical, and management characteristics of previous infants reported with vitamin D intoxication highlighting the course of illness in symptomatic and asymptomatic patients.
