# Supplementary material for: An Infant with Asymptomatic Vitamin D Intoxication: A Prolonged and Sustainable Recovery
Source: Case Rep Endocrinol. 2022 Feb 27;2022:7072815. doi: 10.1155/2022/7072815 (PMC8898842; doi:10.1155/2022/7072815)
Supplement: Supplementary Materials — Figure 1: vitamin D levels per number of weeks over 2.5 years of follow-up. Table 1: biochemical values related to vitamin D metabolism in our patients over 2 years follow-up. Supplement A: clinical, biochemical, and management characteristics of previous infants reported with vitamin D intoxication highlighting the course of illness in symptomatic and asymptomatic patients. [file 7072815.f1.doc]

**Supplement A – Clinical, Biochemical and management characteristics of previous infants**

**reported with Vitamin D intoxication**

|  | **Age** | **Sex** | **Dose Frequency** | **Duration before diagnosis** | **25 OHD level** | **Time to normalize 25OHD** | **Additional remarks** | **Reference** |
| --- | --- | --- | --- | --- | --- | --- | --- | --- |
| **Symptomatic** | One month and a half | Male | 200.000 IU/day vitamin D for 30 days Due to wrong prescription |  | 98 ng/dl (day 8)  266 ng/dl (day 20)  828 ng/dl (day 34) |  |  | [17] |
| **Symptomatic** | 18 months | Male | Multivitamin preparation once daily (50.000 International Unit (IU) of vitamin D and 10.000 IU of vitamin A) for 3 month for wide anterior fontanel | 6 days | 2271 ng/dl |  |  | [18] |
| **Symptomatic** | 5.5 months | Male | 14 400 IU daily x 4 months (mom administered a dropper full or 1mL) |  | 25(OH)D > 150 ng/mL (30-100) |  |  | [19] |
| **Symptomatic** | 4 months | Female | 50 000 IU daily x 2 months (mom administered a dropper full) | 3 days | 25(oh)D 294 ng/mL (30-100) | 89 days |  | [20] |
| **Symptomatic** | 3.5 months | Female | Vitamin D 2000 IU/mL, 1 mL daily for 2.5 months |  | 25 (OH)D 644 ng/mL |  |  | [21] |
| **Symptomatic** | 2.5 months | Male | 200.000 IU vitamin D for 1.5 weeks (prescribed by chiropractor) |  | 25 (OH)D 680 ng/mL |  |  |  |
| **Symptomatic** | 7 months | Male | 1200-1600 IU daily for 4 months | 3 months | Vit D is more than 150 µg/dL |  | Mother was on regular Vit D supplementation pre & post pregnancy as advised by Physician without knowing serum Vit D level. | [13] |
| **Symptomatic** | 2.5 years | Male | Consumption of fish oil supplement containing excessive high dose of Vitamin D. Estimated daily amounts of vitamin D intake varied between 266 000 and 800 000 IU | 2 weeks | 962 ng/mL | 120 days |  | [22] |
| **Symptomatic** | 1.1 years | Male |  | 12 days | 340 ng/mL | 120 days |  |  |
| **Symptomatic** | 1.7 years | Female |  | 3 days | 736 ng/mL | 120 days |  |  |
| **Symptomatic** | 3.2 years | Female |  |  | 620 ng/mL | 120 days |  |  |
| **Symptomatic** | 1.2 years | Male |  |  | 841 ng/mL | 120 days |  |  |
| **Symptomatic** | 4.2 years | Male |  |  | 488 ng/mL | 120 days |  |  |
| **Symptomatic** | 0.7 years | Female |  |  | 467 ng/mL | 120 days |  |  |
| **Asymptomatic** | 1.5 months | Female | 10 drops daily, total of 200 000 IU per month |  | 25(OH)D > 400 nmol/L 50-125) |  |  | [23] |
| **Asymptomatic** | 3 months | Male | 12 000 IU daily x 20 days (mom gave 1 mL) |  | 25(OH)D = 422 ng/mL(30-100) |  |  | [24] |
